# Molecular Design of Magnetic Resonance Imaging Agents Binding to Amyloid Deposits

**DOI:** 10.3390/ijms241311152

**Published:** 2023-07-06

**Authors:** Alena Nikiforova, Igor Sedov

**Affiliations:** Chemical Institute, Kazan Federal University, Kremlevskaya 18, 420008 Kazan, Russia

**Keywords:** Alzheimer’s disease, amyloid plaques, amyloid fibrils, magnetic resonance imaging, molecular imaging probes, fluorine-19, protein-ligand interactions, imaging agent development

## Abstract

The ability to detect and monitor amyloid deposition in the brain using non-invasive imaging techniques provides valuable insights into the early diagnosis and progression of Alzheimer’s disease and helps to evaluate the efficacy of potential treatments. Magnetic resonance imaging (MRI) is a widely available technique offering high-spatial-resolution imaging. It can be used to visualize amyloid deposits with the help of amyloid-binding diagnostic agents injected into the body. In recent years, a number of amyloid-targeted MRI probes have been developed, but none of them has entered clinical practice. We review the advances in the field and deduce the requirements for the molecular structure and properties of a diagnostic probe candidate. These requirements make up the base for the rational design of MRI-active small molecules targeting amyloid deposits. Particular attention is paid to the novel cryo-EM structures of the fibril aggregates and their complexes, with known binders offering the possibility to use computational structure-based design methods. With continued research and development, MRI probes may revolutionize the diagnosis and treatment of neurodegenerative diseases, ultimately improving the lives of millions of people worldwide.

## 1. Introduction

Alzheimer’s disease (AD) is a slowly progressing neurodegenerative disorder that accounts for 60 to 80% of dementia cases [1]. Until recently, a definitive diagnosis of AD could be made only post-mortem, while clinical diagnoses of AD were “possible’’ or “probable” [2,3,4]. They were based on medical history and clinical symptoms, such as memory dysfunction and cognitive impairment, in different intellectual domains [5,6]. However, cognitive impairment and other obvious pathological changes are observed at the late stage, when the neurons are already dead and the condition is impossible to reverse [3]. In 2011, these criteria were updated, and advances in AD imaging research were taken into account [5]. Among the major pathological biomarkers differentiating AD from other forms of dementia are senile β-amyloid (Aβ) plaque deposition and neurofibrillary tangles (NFTs) of hyperphosphorylated tau protein in the brain [4,6,7,8,9]. In accordance with the hypothesis of the amyloid cascade in AD, the formation of senile plaques with subsequent deposition in tissues is the earliest pathological change in the disease. [10,11,12,13]. The study of dominantly inherited AD demonstrated that amyloid plaque deposition in the brains of the participants occurred about 20 years before the first onset of symptoms in the parents of the participants [14]. Thus, Aβ plaques represent the most important early diagnostic indicator of AD. Diagnostic agents visualizing these plaques will help in the early diagnosis of AD; determining the stage of the disease; assessing the response to anti-amyloid therapy, including clinical trials of promising drug candidates; and developing new treatment strategies [3,15].

Positron emission tomography (PET) scan-based diagnostics of amyloid deposits with clinically approved imaging probes demonstrate very high specificity and sensitivity in AD patients. Unfortunately, the use of PET is still limited due to its cost, as it is not covered by most insurance companies, so it is still used as part of clinical trials [6]. In addition, PET has a low spatial resolution, not allowing for the visualization of individual plaques [16]. Considering the severity of the disease and the increasing incidence rate of AD [1,17,18], the development of other affordable and effective diagnostic methods is relevant. The alternatives to radio imaging are optical imaging and magnetic resonance imaging (MRI) techniques, which can also use chemical probes that are able to bind to Aβ plaques or NFTs.

In this review, we focus on the recent progress in the development of novel MRI diagnostic agents binding to fibrillar structures. For a more general description of various AD imaging approaches, readers may refer to the reviews of Kaur et al. [19], Arora and Bhagat [20], and Mori et al. [21]. Some previous papers were also dedicated particularly to AD radioimaging diagnostic agents [22,23,24,25,26,27,28] and fluorescent probes [29,30,31,32,33]. Various aspects and techniques of MR imaging of AD which do not focus on the fibril-binding tracers have also been reviewed [34,35,36]. In contrast, herein, we highlight the strategies and prospects for the molecular design of compounds with desirable properties for MRI tracing. Some of these properties, such as a high affinity or blood–brain barrier (BBB) permeability, are also important for other imaging techniques, which leads to similar structural patterns being observed in optical, radio, and MR imaging probes. At the same time, each method has its own specific requirements for the probe structure. MRI relies on the presence of spin-active nuclei in the probe. In addition, when the probe is bound to the amyloid plaque, a strong signal can be only obtained if the segment containing the active nucleus is sufficiently flexible.

## 2. Major Amyloid Visualization Techniques

### 2.1. Optical Imaging

**Figure 1:**
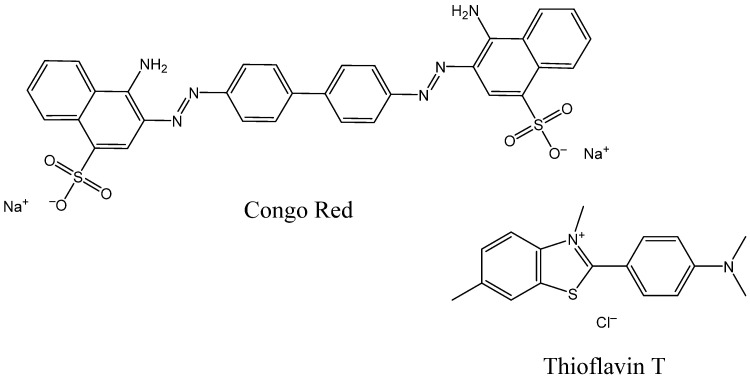
Structures of Congo Red and Thioflavin T.

Fluorescent probes are widely used for in vitro and ex vivo studies of soluble and insoluble Aβ aggregates. Optical methods also have some potential for in vivo studies, with the advantages of low cost, ease, and speed of analysis [29]. The first dyes applied in histopathological studies of amyloidosis were two fluorescent indicators: Congo red, which has been used since 1922, and Thioflavin T, used since 1959 (Figure 1). They are still frequently chosen for immunostaining of brain tissues for the post mortem diagnosis of AD and in in vitro studies of fibril formation kinetics [37,38,39,40,41].

However, in vivo imaging requires deep tissue penetration and a good signal-to-background ratio, so UV/visible fluorescent probes cannot be used. Near-infrared fluorescence (NIRF) has become the most widely used in vivo optical imaging method. It is based on the detection of photons with 650–900 nm wavelengths emitted by the excited fluorophores. Near-infrared light has deeper tissue penetration compared to visible light due to less absorption by hemoglobin and water and less autofluorescence from surrounding tissues [42]. Unfortunately, even NIRF is suitable only for superficial imaging of organ tissues in humans [42]. The quality of deep tissue imaging is far from adequate, and the depth of penetration of NIR photons into tissues is only several millimeters [43,44]. NIRF is useless in the case of AD due to weak penetration of light into the skull-shield brain. Imaging of amyloid deposits in the brains of small animals is possible, but often requires skull thinning to achieve satisfactory image resolution [30]. Moreover, undesirably high concentrations of fluorescent probes would be required for in vivo studies due to their low sensitivity.

**Figure 2:**
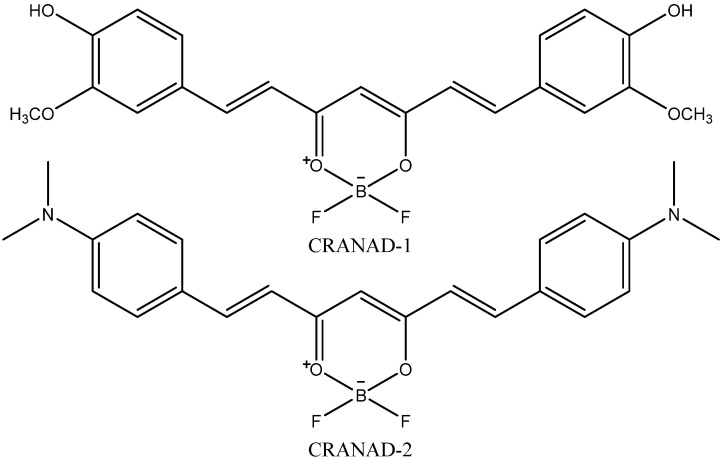
Structures of CRANAD-1 and CRANAD-2.

**Figure 3:**
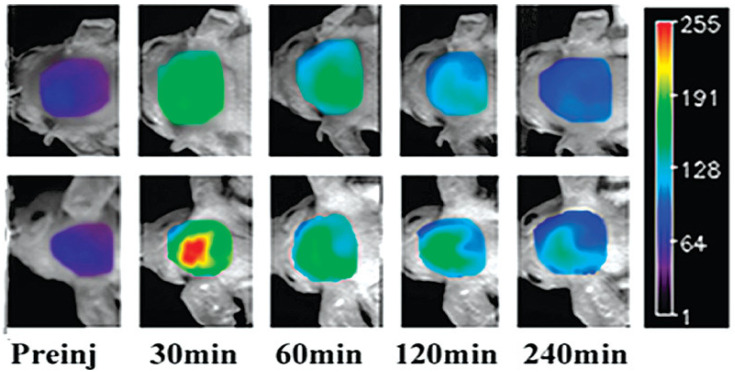
NIR fluorescence images of the brains of wild-type (top) and Tg2576 AD model mice at different time points, before (Preinj) and after injection of 5.0 mg/kg CRANAD-2. Adapted with permission from [45]. Copyright 2009 American Chemical Society.

One of the prospective NIR fluorescent probes for amyloid aggregates is CRANAD-2. Its synthesis [45] was inspired by the knowledge that incorporating a difluoroboronate moiety into yellow-colored curcumin, which is able to bind Aβ fibrils, leads to red dye (CRANAD-1, Figure 2) with higher absorption and emission wavelengths. The introduction of the red-shift pushing N,N-dimethyl groups into the curcumin scaffold led to CRANAD-2 (Figure 2), which also had a good BBB-penetrating ability. Figure 3 shows in vivo brain images created using CRANAD-2 injected into mice. The development of fluorescence molecular tomography (FMT) and integration with other imaging techniques may offer novel opportunities for fluorescent indicators. For example, Ren et al. [46] demonstrated the ability of FMT integrated with MRI to visualize 3D deposition of beta-amyloid in mouse brains using CRANAD-2.

**Figure 4:**
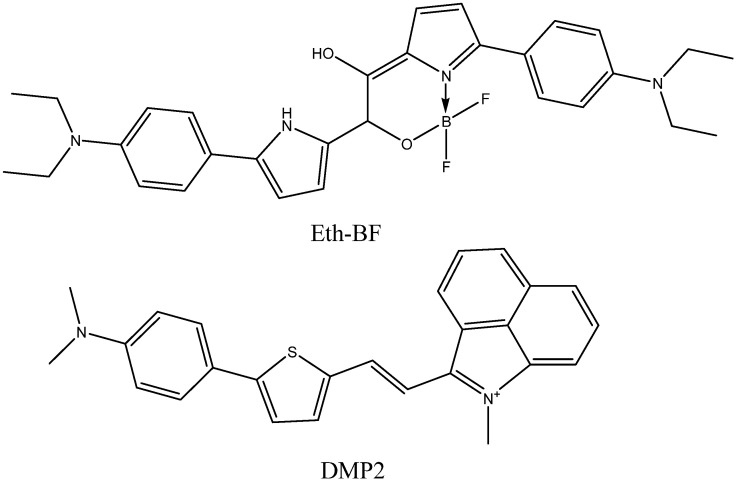
Structures of Eth-BF and DMP2.

Further progress in fluorescence bioimaging may also be connected with NIR-II fluorescent probes emitting in the 950–1350 nm wavelength range [43]. NIR-II light has a larger (centimeter-level) penetration depth and allows for micron-level resolution to be obtained. However, few NIR-II probes have been developed. Recently, a difluoroboronate Eth-BF (Figure 4) was synthesized and tested in mice [47]. It contains N,N-diethylaniline fragments presumed to bind to amyloid fibrils. They also serve as donor groups, leading to the strong intramolecular charge transfer effect, which results in high emission wavelengths with emission tails reaching 1200 nm. Another NIR-II probe shown to visualize amyloid plaques in mice, DMP2 (Figure 4), with 980 nm emission maximum and 42 nM affinity to fibrils, is a conjugate of N,N-dimethylaminobenzene, thiophene, and benzo[cd]indole-1-ium fragments [48].

Nevertheless, decades of efforts aimed at the design of fluorescent probes for amyloid aggregates has led to the discovery of different molecular scaffolds providing a good affinity to fibrils. The compounds developed to be used as histological dyes, for visualization of fibril formation in vitro, or NIR fluorescent probes can be turned into MRI or radio imaging probes after some modifications introducing the necessary isotopes. We discuss the molecular structure of known fibril binders in more detail in Section 3.

### 2.2. Radio Imaging

Radio imaging, which includes positron emission tomography (PET) and single photon emission computed tomography (SPECT), is based on the detection of gamma rays from the decay of certain isotopes in the probe molecule.

PET is a powerful imaging technique for detecting Aβ fibril deposits with very low doses of radioactive indicators [49]. PET indicators most commonly contain the ^11^C, ^18^F, ^64^Cu, or ^68^Ga isotopes. These radionuclides contain excess amounts of protons, are unstable, and undergo radioactive decay by emitting positrons. Positrons collide with nearby electrons and slow down and ultimately annihilate them, either in flight or at rest, to emit two 511 keV gamma rays in opposite directions. The instrument identifies coincident gamma pairs and determines, with high accuracy, the position of the line on which the annihilation process occurred [23,50,51,52]. SPECT indicators usually contain ^123^I, ^125^I, ^99m^Tc, ^111^In, or ^188^Re isotopes, the nuclei of which emit one gamma photon during each act of radioactive decay. These photons leave the patient’s body unscattered and can also be used to obtain information about the distribution of the radiopharmaceutical in the organs and tissues [23,52,53,54].

**Figure 5:**
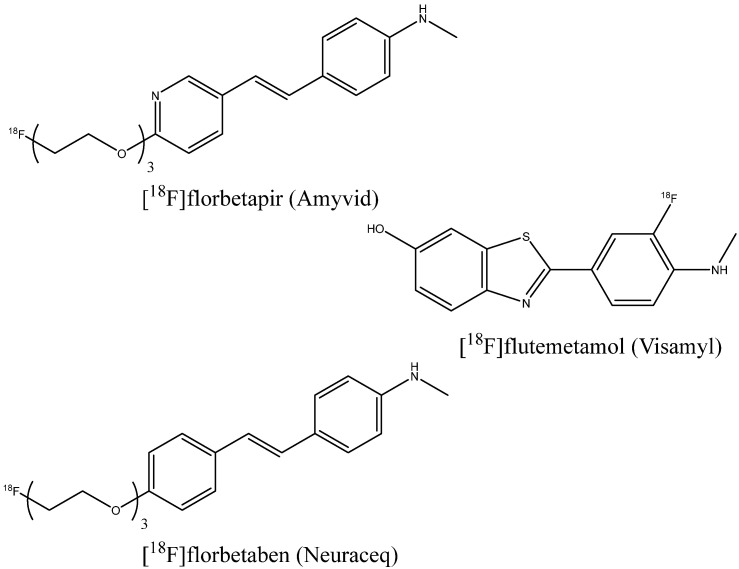
FDA- and EMA-approved PET imaging indicators.

**Figure 6:**
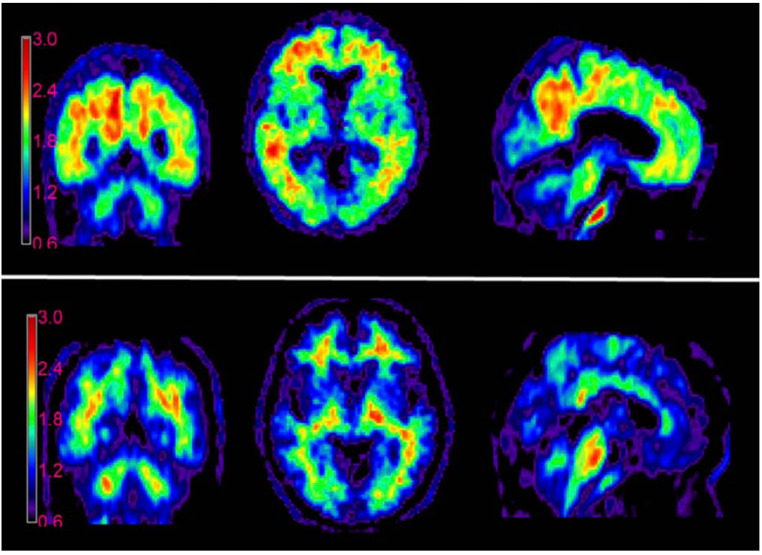
PET images of AD-affected (**top**) and healthy (**bottom**) human brains, made using [^18^F]florbetapir. This figure was originally published in JNM (Ref. [55]). © SNMMI.

The U.S. Food and Drug Administration (FDA) and the European Medicines Agency (EMA) have approved several indicators for PET imaging of beta-amyloid in patients with cognitive impairment undergoing clinical assessment for AD: [^18^F]florbetapir (Amyvid) (2012) [55], [^18^F]flutemetamol (Vizamyl) (2013) [56], and [^18^F]florbetaben (Neuraceq) (2014) [57] (Figure 5). For [^18^F]florbetapir, 59 post mortem brain assays were performed, showing sensitivity and specificity of 92% and 100%, respectively [58]. For 74 assays with [^18^F]florbetaben, the sensitivity and specificity were 97.87% and 88.89%, respectively [59], and for 106 patients with [^18^F]flutemetamol, these values were 85.7% and 100% [60]. Figure 6 shows an example of the PET images of healthy and AD-affected human brains after injection of [^18^F]florbetapir.

**Figure 7:**
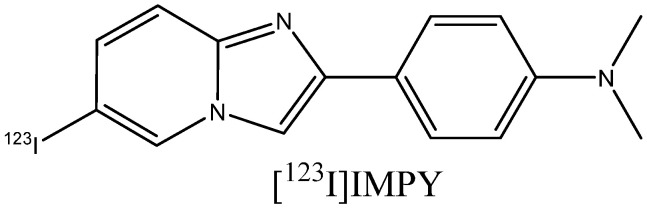
Structure of [^123^I]IMPY.

The inclusion of the ^18^F label into PET indicator molecules occurs through nucleophilic radiofluorination by ^18^F– ion, which can be produced via the ^18^O(p,n)^18^F reaction when ^18^O-enriched water is irradiated with protons in cyclotrons [61,62,63]. Due to the short half-life of isotopes for the synthesis of PET and SPECT indicators, synthesis should be designed with a minimum number of steps and purification, in order to take the shortest possible amount of time [64]. The ^18^F isotope has a half-life of 109.8 min [65]. This is longer than that of the isotope ^11^C (20 min) used in some earlier developed indicators, which allows for a reduction in the cost of the indicator and an increase in the number of potential users of imaging centers [55]. The isotopes used in SPECT have longer half-lives, but technetium, indium, and rhenium can be only infused in the form of complexes, which usually have poor BBB permeability [19]. A number of clinical trials have been conducted with ^123^I-containing probes, e.g., [^123^I]IMPY [66] (Figure 7), which displayed a low signal-to-noise ratio. No SPECT amyloid probes have been approved for clinical use.

Besides the radiation risk, the cost and complexity of synthesis, and the purification of diagnostic agents, the disadvantages of PET imaging are quite low resolution and high background noise resulting from the nonspecific absorption of ^18^F-indicators by the white matter of the brain [19,67]. The disadvantages of SPECT are even lower spatial resolution, long scan times, and difficulty in obtaining reliable quantification [68].

### 2.3. MR Imaging

MRI is based on the effect of nuclear magnetic resonance relaxation. The conventional ^1^H MRI technique is based on the relaxation of water proton spins in the human body. A number of metal-based (mainly gadolinium-based) contrast agents have been developed which increase the relaxation rate in the immediate vicinity of the metal and improve the visibility of pathologies [69]. Conventional or contrast-enhanced ^1^H MRI has been successfully used to diagnose many different pathological conditions: neurological tumors [70]; issues with the structure and function of the heart [71,72]; joint diseases and soft tissue tumors [73,74,75,76]; stenosis or aneurysm of the arteries [77]; and lesions of the liver, pancreas, and bile ducts [78,79,80].

In AD-suspected patients, MRI allows for non-invasive measurement of the brain tissue volume, which helps to localize pathophysiological signs such as regional brain atrophy, gray matter volume loss, and white matter damage [15,34,35,81]. However, these signs are not specific signatures of AD, and the number of brain cells or white matter tracts that should be lost to detect atrophy in AD is unknown [15]. Direct visualization of amyloid deposits is a different and prospective approach. Unfortunately, the ability to identify Aβ plaques is very limited, even in the presence of contrast agents [82]. One possible solution is the development of targeted contrast agents with a binding affinity to plaques, which are discussed below in Section 3.3. The common problem of such agents is low BBB permeability. In addition, ^1^H MRI does not directly detect contrast agent molecules, and the signal is not proportional to their concentration [83].

**Figure 8:**
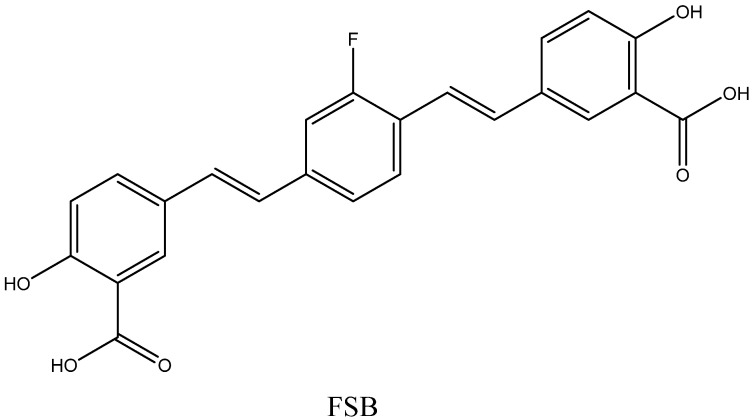
Structure of FSB.

Another method is the ^19^F MRI technique, with fluorinated organic probes binding to amyloid fibrils. Multi-nuclear imaging is more challenging than ^1^H imaging and requires very sensitive radiofrequency coils tuned to specific frequencies, broadband radiofrequency amplifiers, and specialized pulse sequences [84]. However, the ^19^F MRI method has a number of advantages. The sensitivity of the magnetic resonance of ^19^F is relatively high compared to the sensitivity of various nuclei other than ^1^H [85,86,87]. The natural abundance of ^19^F is 100%, which means that no isotope-enriched reagents nor radioactive compounds should be used during probe synthesis. At the same time, there is almost no fluorine in the human body, and the background noise is very low [88]. Unlike the indirect effect of metal-based contrast agents on the water signal in ^1^H MRI, the detection of fluorine-based agents directly visualizes ^19^F atoms, and the signal is proportional to their quantity [89]. In the pioneering work of Higuchi et al. [90], (E,E)-1-fluoro-2,5-bis-(3-hydroxycarbonyl-4-hydroxy)styrylbenzene (FSB) probe (Figure 8) was used to detect Aβ plaques in mouse brains. This compound was originally synthesized as a fluorescent dye with amyloid-binding ability and good BBB permeability [91]. Further progress in fluorinated MRI probe development is discussed in Section 3.1. Not all of these probes, however, have reached the clinical phase yet.

The general advantages of MRI include relatively high spatial resolution, low cost (about one-fifth of the PET cost), wider availability, use of diagnostic agents with a long shelf life, and the absence of ionizing radiation [19,90]. Due to the high resolution, this method can provide anatomical information, helping to identify the brain regions with amyloid deposits and quantitatively characterize the extent of deposition [83]. The main disadvantage is the low sensitivity of the MRI method in general. This can be improved by designing fluorinated probes with strong signals, which is a specific requirement for the ^19^F MRI diagnostic agents, as described below in Section 3.1.

## 3. Molecular Design of MRI Fibril-Binding Probes

### 3.1. Fluorinated Probes Binding to Amyloid Plaques

**Figure 9:**
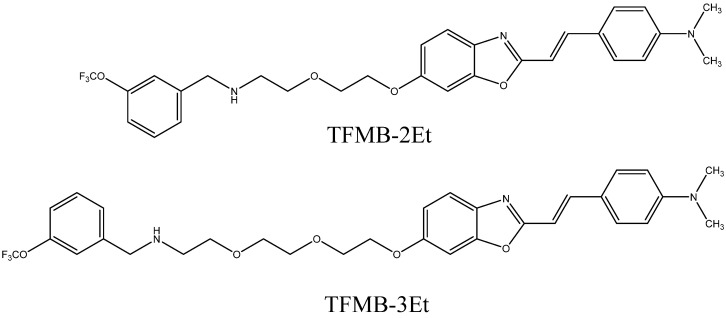
Structures of TFMB-2Et and TFMB-3Et.

The low signal-to-noise ratio was the main problem of the FSB probe [90]. In order to increase it, the authors suggested increasing the number of fluorine atoms in the probe molecule and its affinity to amyloid aggregates. Vennerstrom et al. [92] prepared tetrafluorosubstituted and trifluoromethylsubstituted bis-styrylbenzenes. These compounds had higher affinities to beta-amyloid than FSB and four to six fluorine atoms in their molecules, but had not been tested in vivo. Tooyama et al. studied benzoxazole compounds with the trifluoromethoxy groups TFMB-2Et and TFMB-3Et [93] (Figure 9). These compounds showed a strong ^19^F signal in a buffer, but it was greatly reduced in mouse brain lysates. A similar reduction was also observed when the NMR spectra of some arbitrary non-amyloid binding fluorinated compounds were recorded in brain homogenates instead of a buffer. The change in signal intensity was smaller for hydrophilic than for lipophilic compounds. The authors attributed this reduction to the high membrane lipid content in the brain, particularly in myelin sheaths. Lipid components may trap and immobilize lipophilic compounds, leading to short relaxation times and broadened NMR peaks. On the other hand, hydrophilic compounds face difficulties with BBB penetration. Thus, it is necessary to provide optimal lipophilicity to the MRI probes. Other imaging techniques also suffer from the nonspecific binding of lipophilic compounds. Approved PET probes, florbetapir, and flutemetamol contain a polyethylene glycol chain, which increases their hydrophilicity.

**Figure 10:**
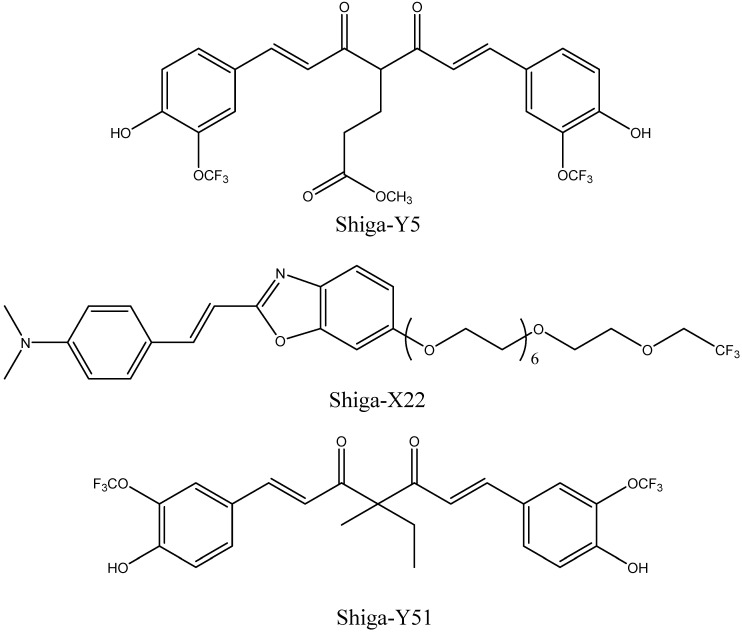
Structures of Shiga-Y5, Shiga-X22, and Shiga-Y51.

**Figure 11:**
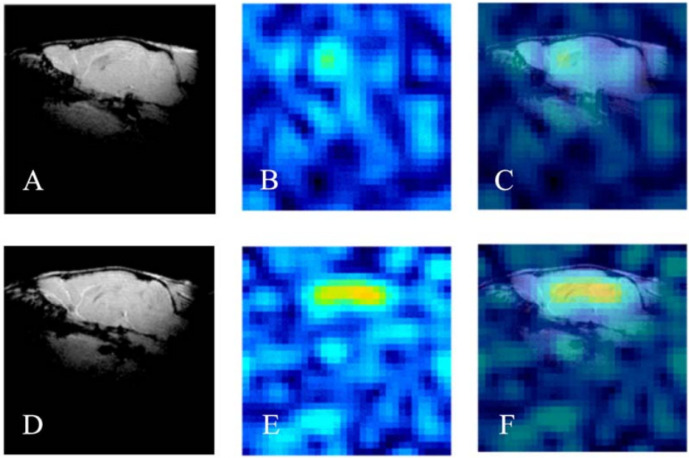
^1^H (**A**,**D**), ^19^F (**B**,**E**), and merged MR in vivo images (**C**,**F**) of the brains of wild-type (WT, **A**–**C**) and transgenic (APP/PS1, **D**–**F**) mice injected with Shiga-X22 probes. Reprinted from [67] with permission from Elsevier.

**Figure 12:**
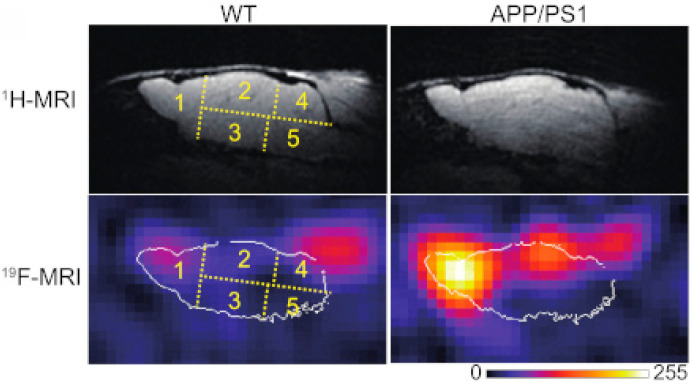
Representative ^1^H and ^19^F MR in vivo images of the brains of wild-type (WT) and transgenic (APP/PS1) mice, obtained 100 min after the injection of Shiga-Y51 probes. Numbers 1−5 indicate different brain regions. Image adapted from [97].

The same research group developed ^19^F-containing curcumin-derivative FMeC1 (also called Shiga-Y5, Figure 10) [94,95] with a high affinity of the enol form to Aβ aggregates, which displayed an MRI signal in a live mouse model. Very interestingly, the authors observed no signal when the brain was resected and immediately subjected to MRI measurements. After several hours, however, the signal from the isolated brain could be registered. This was explained by the hypothesis that binding of the probe to amyloid plaques also leads to the immobilization and NMR signal broadening and disappearance, and the observed signal corresponds to the free probe molecules around the plaques and in the blood. In order to prevent such a signal reduction, it has been suggested that the mobility of ^19^F atoms be increased by separating them, using a flexible polyethylene glycol chain, from the part of the molecule that binds to the amyloid plaques [67,96]. The probes were not well-soluble in water, and the surfactants were added for their intravenous injection. The optimum number of ethylene glycol groups needed to achieve the strongest MRI signal was determined and found to be seven (compound XP7 or Shiga-X22, Figure 10). Figure 11 shows in vivo MR images of wild-type and transgenic AD model mouse brains after injection of this compound. Shorter chains led to weaker signals likely due to insufficient fluorine mobility, while compounds with longer chains faced difficulties in BBB penetration. An increase in the number of fluorine atoms, e.g., by introducing two trifluoromethyl groups instead of one, does not lead to stronger signals, which can be explained by a higher affinity to brain lipids. Another candidate compound in this series is Shiga-Y51 [97] (Figure 10), a fluorinated curcumin derivative with keto-groups not capable of enolization. It does not have a flexible chain with fluorine atoms. Instead, it has been reported to bind soluble Aβ oligomers in the brain, which are significantly mobile and do not cause a dramatic decrease in the relaxation time or the signal intensity. However, strong and unwanted signals were observed even in the experiments with healthy mice. Figure 12 shows the results of in vivo testing of this compound in mice.

**Figure 13:**
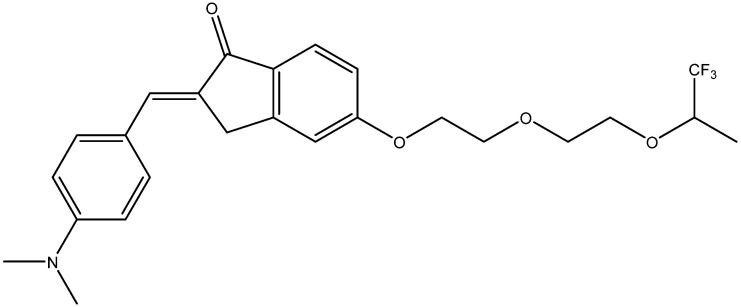
Structure of 7d compound [98].

Recently, a series of potential ^19^F MRI probes was synthesized from indanone derivatives [98]. They contained trifluoromethyl groups separated from the amyloid-binding fragment by two ethylene glycol groups. One of them, abbreviated as 7d (Figure 13), has shown imaging capability in in vitro experiments.

An attempt to use bovine serum albumin-capped graphene quantum dots functionalized with hydrofluorinated glucose as MRI probes in live mice has been made [99]. Graphene quantum dots have an affinity to amyloid peptides, and albumin prevents them from aggregation and reduces their interactions with plasma proteins.

To summarize, ^19^F-MRI can become a readily available and cost-effective approach once sufficiently sensitive probes have been developed. The developed compounds cannot provide the desired signal intensity, even though MR scanners with high magnetic power (7.0 to 9.4 T) were used in the studies described above [95,98]. The sensitivity must also be improved for safety reasons, since quite high doses of probes (e.g., 200 mg/kg [67]) were used, which are likely to be toxic and resulted in the death of mice in some cases. In addition, low sensitivity leads to very long image acquisition times of up to several hours. Hence, novel compounds need to be designed, screened, and tested in vivo.

#### 3.1.1. Requirements for an MRI Probe Candidate

**Figure 14:**
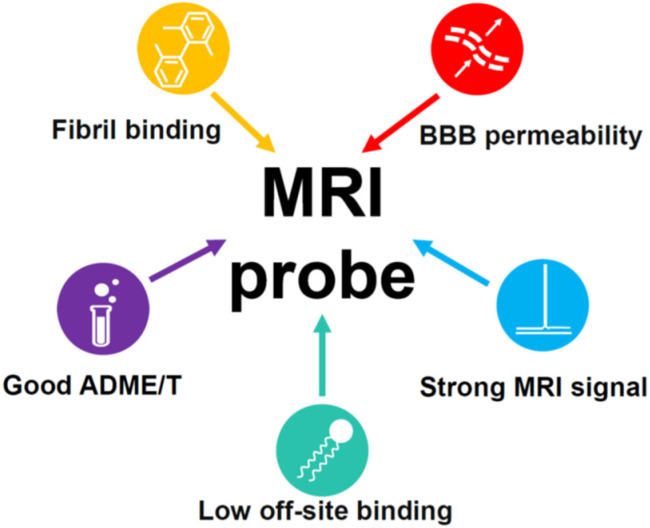
Schematic representation of the main requirements for an MRI probe candidate.

The requirements that an MRI probe candidate must meet are as follows (see Figure 14 for a short schematic representation):

1. High affinity and specificity of binding to Aβ plaques: The affinity is usually determined in vitro using beta-amyloid binding assays based on radioligand binding, fluorescence titration, or other techniques. These experiments are not robust, and the value for the same compound may vary significantly depending on the exact conditions and methods used by different authors, which complicates our understanding of the structure–affinity relationships. The presence of certain molecular scaffolds discussed in the next section can guarantee a certain degree of affinity to beta-amyloid, which can be enhanced by various structural modifications. The approved PET probes have an affinity below 10 nM, and most of the compounds studied as potential probes for various imaging techniques have an affinity in the range of 1 to 1000 nM. The introduction of long, flexible chains carrying fluorinated groups into the molecule is likely to result in a decrease in the affinity [100]. Hence, the design of MRI probe candidates should be initiated from the precursors with the highest possible amyloid affinity.

The specificity of binding can only be proven experimentally. The approved PET probes are known to bind highly specifically; hence, the structurally similar compounds will also likely be specific. It is especially difficult to provide the selectivity for amyloid plaques over neurofibrillary tangles, which consist of tau protein aggregates with similar β-sheet structures. On the other hand, some compounds have been developed which bind tau aggregates more tightly than beta-amyloid plaques and can be used to diagnose tau pathologies (see Section 3.2.1).

2. Blood–brain barrier (BBB) permeability: The ability of amyloid probes to cross the BBB is indispensable for MRI imaging in vivo. Passive transmembrane diffusion of a compound through the BBB is possible for rather small lipophilic molecules. A molecular weight between 400 and 600 Da is usually considered as an upper border for BBB-permeable compounds. Under this threshold, the kinetic permeability logPS and the steady-state blood–brain partition coefficient logBB are, to some extent, correlated with 1-octanol-water partition coefficient logP (or logD for ionizable compounds) [101]. For larger molecules, the passive permeability rapidly decreases with the molecular size and is generally not correlated with logP. The cutoff value of logBB = 0 can be taken to classify compounds as permeable, which means an equal concentration of a compound on both sides of the barrier. The equivalent cutoff value for logPS is about –2 [101]. Despite the fact that the relationship of these quantities with logP is not strict, the value of logP (logD) > 1 is often recommended for the molecules that should pass the BBB. Additional commonly mentioned empirical rules for the BBB-permeable compounds are to form no more than eight hydrogen bonds with water; not to carry a negative charge, since the surface of the brain endothelial cells forming the BBB is itself negatively charged; and not to be a high-affinity serum protein binder [102,103]. For a more precise a priori logBB prediction, multiple QSAR models based on linear regressions or various machine learning approaches [104,105,106] have been developed.

Active targeting strategies include the use of adsorptive-mediated transcytosis, transporter-mediated transcytosis, and receptor-mediated transcytosis [107]. These mechanisms allow for the brain delivery of some large or hydrophilic molecules necessary for organism function. The MRI probes that cannot cross the BBB themselves can be encapsulated into or conjugated to nanoparticles modified with specific ligands that are recognized by the receptors or transporters, or coupled to BBB-penetrating proteins [107,108,109].

3. Low binding to the membrane lipids in the brain: Binding to membrane lipids is thought to decrease the signal-to-noise ratio and can be reduced by lowering the probe’s lipophilicity. The upper border of the optimal logP (logD) value is about 3–4. The approved PET probes are moderately lipophilic, i.e., logD = 1.58 for florbetaben [110] and logP = 3.44 for flutemetamol [111], while for Shiga-X22, the predicted logP value equals 3.77 [67].
4. Other ADMET properties: Sufficient in vivo stability, low toxicity, and an optimal clearance rate are necessary for the successful use of a compound as a diagnostic agent. Most of the known probe candidates have been studied in live mice and cell cultures and found to have low toxicity, with the exceptions of some compounds with long ethylene glycol chains [67] and possible nephrotoxicity of FSB at high doses [90].
5. Strong MRI signal. The NMR signal intensity of a compound is proportional to the number of fluorine atoms in its molecule [112,113]. The MRI probes that were studied in vivo contained one or two trifluoromethyl groups, and their signal-to-noise ratio was far from desirable [67,96]. However, as mentioned above, a larger number of fluorine atoms leads to higher hydrophobicity and increased off-target interactions with brain lipid components, as well as low solubility in water. An alternative strategy could be a simultaneous introduction of fluorines and polar groups, decreasing the hydrophobicity of a probe. The fluorine atoms should be arranged symmetrically in order to provide a single NMR peak. Moreover, fluorines should be separated from the amyloid-binding fragment by a flexible chain to avoid signal broadening and disappearance.

**Figure 15:**
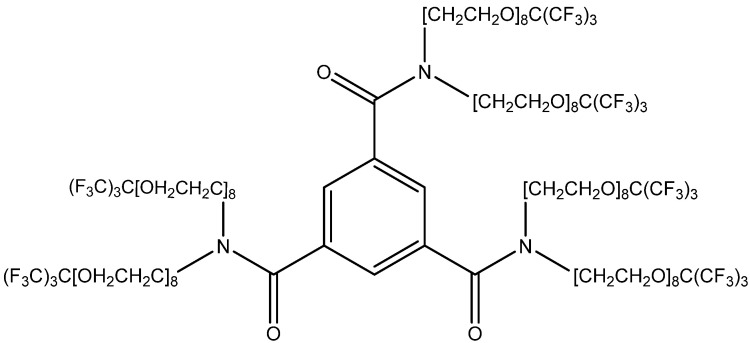
Structure of a water-soluble MRI-traceable compound (41) with 54 fluorine atoms [115].

A number of dendrimeric compounds containing a high number of equivalent fluorine atoms have been synthesized recently, some of which are water-soluble (an example is shown in Figure 15) [114,115]. They can be conjugated with the amyloid-binding scaffolds discussed below. Such molecules, of course, will face problems with BBB permeability and require some special transport technologies, e.g., nanoparticle-based ones [115].

#### 3.1.2. Molecular Fragments Providing Affinity to Aβ Fibrils

The structural features of beta-amyloid fibrils, as well as fibrils from other proteins, are stacked parallel arranged β-sheets with a tendency of the hydrophobic residues to be packed within the fibril and the hydrophilic residues to be water-accessible [116,117]. Experimental and theoretical studies indicate the binding of ligands to several binding sites in different spatial orientations [27] and the existence of tightly and loosely bound ligand molecules [118]. In addition, bound ligands in general do not obey the helical symmetry of fibrils. Thus, the exact binding poses can be inaccessible even for cryo-EM studies [119]. Nevertheless, the combination of cryo-EM or NMR data with docking and molecular dynamics simulations provides information about the binding sites and ligand orientation.

**Figure 16:**
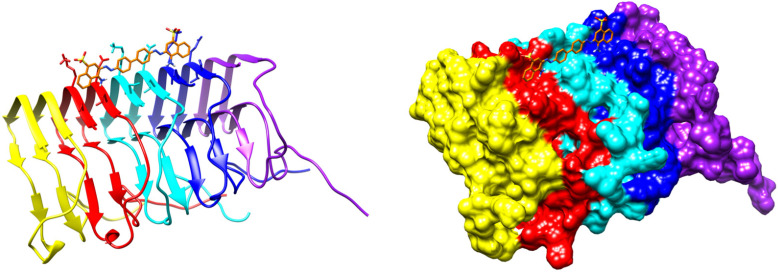
Two representations of the result of docking of Congo Red to the prion domain of the fungal HET-s protein with NMR restraints [121]. PDB ID: 2LBU.

In particular, it was shown that a PET probe flutemetamol binds to the surface of Aβ40 fibrils in a planar conformation with the ligand long axis (connecting benzothiazole and benzene rings, Figure 5) predominantly parallel to the fibril axis [118]. This ligand is involved in interactions with polar and aromatic residues. Purely computational studies also predict binding of Thioflavin T, Congo Red, and their derivatives in parallel to the beta-amyloid fibril axis [27,120]. More experimental data on fibril binding are available for proteins other than beta-amyloid. Docking of Congo Red to the prion domain of the fungal HET-s protein with restraints derived from NMR chemical-shift perturbations and polarization transfer experiments [121] results in a planar ligand molecule embedded in a surface groove (Figure 16). The complex is stabilized by electrostatic and hydrogen-bonding interactions with polar residues.

**Figure 17:**
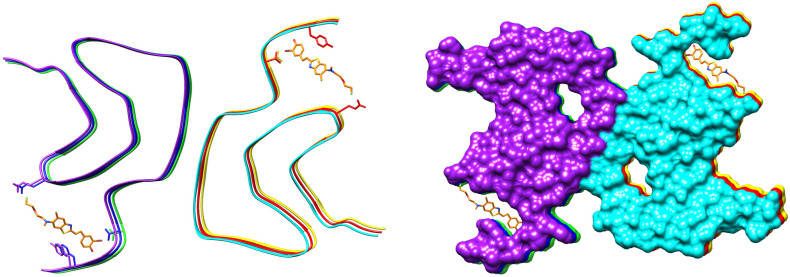
The cryo-EM structure of a complex of fluorinated benzothiazole derivative with α-synuclein fibrils. PDB ID: 7WMM.

The cryo-EM structure of a complex of fluorinated benzothiazole derivative with α-synuclein fibrils available in PDB (7WMM) indicates the accommodation of the ligand in a cavity formed between the beta-sheets (Figure 17). A recent study of interactions of diphenylpyrazole anle138b, a clinical drug candidate, with α-synuclein fibrils has shown binding deep inside this cavity [122]. The molecule of anle138b is relatively small and has no charged or hydrogen-bond donor groups.

**Figure 18:**
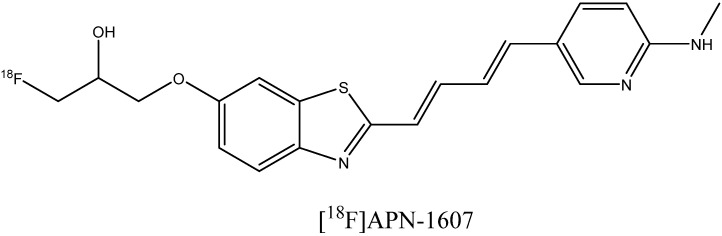
Structure of the PET probe candidate [^18^F]APN-1607.

**Figure 19:**
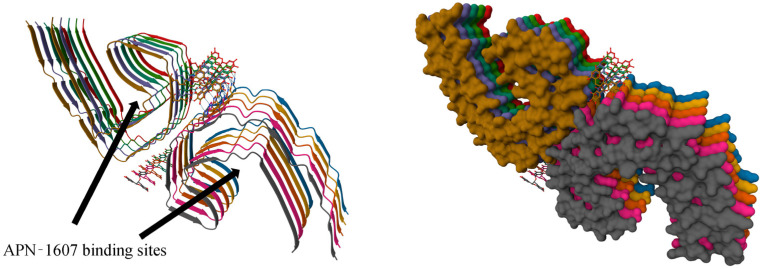
The cryo-EM structure of a paired helical filament of tau protein from an AD-affected brain in complex with epigallocatechin gallate. PDB ID: 7UPG. Arrows indicate the binding location of the APN-1607 ligand according to a different study [119].

The binding of the fluorinated PET probe candidate APN-1607 (Figure 18) with tau protein fibrils was investigated using cryo-EM [119]. This revealed several binding sites inside the C-shaped cavity of tau filaments (see Figure 19). Some bound molecules are parallel, while others are perpendicular to the fibril axis. Minor binding sites in the clefts between two protofilaments have also been reported. In another cryo-EM study, a fibril formation inhibitor epigallocatechin gallate was shown to form parallel stacks in these clefts, contacting polar protein residues (Figure 19) [123]. Stacked binding to the C-shaped cavity of certain types of tau filaments with a molecular stoichiometry close to 1:1 was recently observed for the approved PET tau imaging agent flortaucipir [124] and a tracer candidate GTP1 [125]. In both cases, the π–π stacking aromatic rings formed a 45-degree angle with the helical axis.

Despite the absence of a well-defined binding site in amyloid fibrils, it is possible to deduce some desirable structural features of amyloid binders. A high affinity is expected for the molecules with conjugated aromatic fragments that are able to adopt a planar conformation, preferably with a linear arrangement of aromatic systems and favorable interactions with polar amino acid residues. A number of scaffolds listed below have been shown to possess amyloid-binding properties. They can be modified by various functional groups to tailor affinity and any other properties.

**Figure 20:**
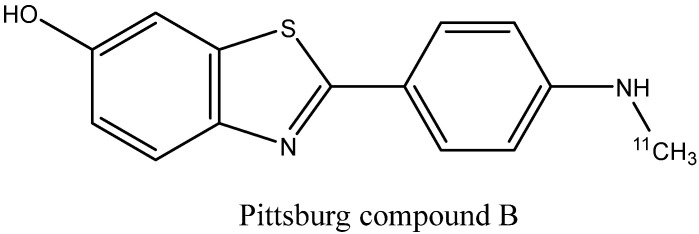
Structure of Pittsburgh compound B.

**Figure 21:**
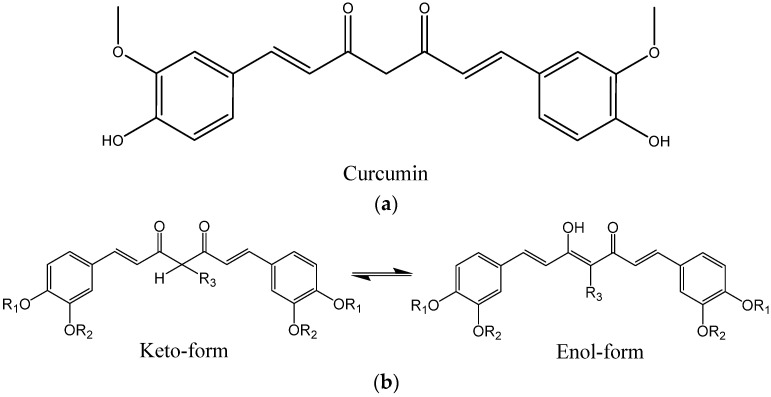
(**a**) Structure of curcumin. (**b**) Keto-enol tautomerism of curcumin derivatives.

**Figure 22:**
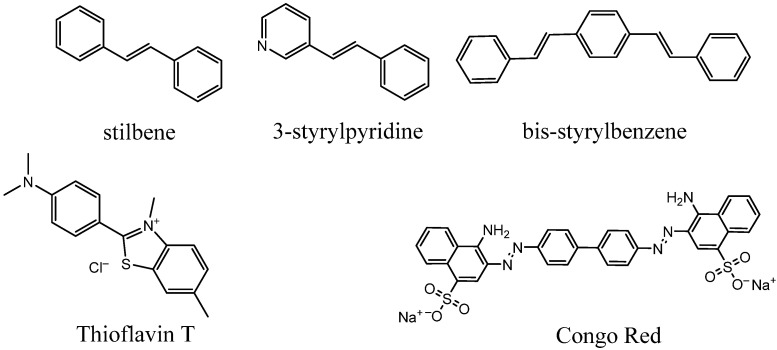
The scaffolds of stilbenes, styrylpyridines, and bis-styrylbenzenes.

**Figure 23:**
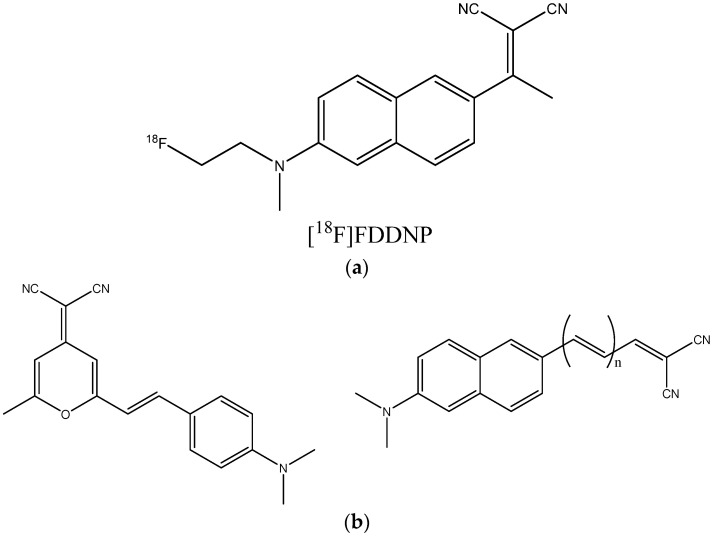
(**a**) Structure of [^18^F]FDDNP. (**b**) Examples of dicyanomethylene derivatives showing their affinity to beta-amyloid fibrils [30].

1. Benzothiazole derivatives [30,126,127]: Prominent representatives are thioflavin T, flutemetamol, and Pittsburgh compound B (Figure 20) [128], a PET probe used in numerous early in vivo imaging experiments [129]. Most of the synthesized ligands contain an aromatic substituent or a conjugated double-bond system attached to the C2 atom. QSAR analysis shows that the monoalkyl amino group in the para position to benzothiazole increases the binding affinity [28]. Substituted benzoxazoles, benzothiophenes, benzofurans, imidazopyridines (e.g., IMPY), and other bioisosteric heterocycles [130,131] can also show strong fibril binding.

2. Curcumin (Figure 21a) derivatives [45,132,133,134], which include CRANAD dyes and some of the Shiga-Y series compounds: It was reported that the enol form of curcumin derivatives (Figure 21b) exhibits a high affinity to Aβ fibrils, while the keto form shows weaker binding, leading to the bound state becoming predominantly enolic [94]. While curcumin itself is chemically unstable, weakly soluble in water, prone to non-specific binding with many different targets, and has a poor pharmacokinetic profile, some of its derivatives can be more suitable for probe development. The replacement of β-diketone with a difluoroboronate moiety in the CRANAD series or with a single carbonyl group [132] helps to overcome the pharmacokinetic limitations. Replacing the two phenyl rings of curcumin with pyridyls can strengthen the interaction with proton donor residues of Aβ [135]. The presence of a rather rigid linker between the aromatic rings, like in curcumin, seems to be a prerequisite for high Aβ affinity [136].

3. Stilbenes, styrylpyridines, and bis-styrylbenzenes (Figure 22) [92,100,137,138]: These compounds also have aromatic rings separated by rigid conjugated double bonds. The aforementioned PET probes, florbetaben and florbetapir, as well as the FSB compound, belong to this group. The studies of their amyloid affinity were inspired by certain structural similarities of stilbenes with benzothiazoles (particularly thioflavin T) and bis-styrylbenzenes with Congo Red dye. Some non-stilbene (azo) fibril-binding Congo Red derivatives have also been obtained [38,139,140]. Bis-styrylbenzenes have also been modified by replacing the central benzene unit with naphthalene, thiophene, or other heterocyclic moieties [137].

4. Dicyanomethylene derivatives [30,141,142]. A well-known representative is the PET probe FDDNP (Figure 23a) [143], used for research purposes. These compounds are π-conjugated systems comprising double bonds and an aromatic ring, often with an electron donor dialkyl amino substituent in para-position (Figure 23b).

5. Other scaffolds: Some carbazole derivatives [144,145], substituted phenothiazines [146], coumarin derivatives [147,148,149], merocyanine [150,151], and hemicyanine-based [152] dyes have been reported to have an affinity to beta-amyloid fibrils.

### 3.2. Probes Binding to Non-Aβ Fibrils

#### 3.2.1. Imaging Tracers of Tau Pathology

In addition to the formation of amyloid plaques, AD has another hallmark: the accumulation of an abnormal, hyperphosphorylated form of tau protein as insoluble neurofibrillary tangles in the brain. Several other neurodegenerative diseases lead to the formation of similar tau aggregates (but not beta-amyloid fibril plaques), and are known as tauopathies. The tangles comprise large fibrillar aggregates dominated by parallel beta-sheets assembled into paired helical or straight filaments. Recent cryo-EM studies [153,154,155,156,157,158] have shown that these aggregates can adopt different conformations depending on the disease or in vitro growth conditions [155,159]. The amount and distribution of neurofibrillary tangles in the brain are known to depend on disease progression.

**Figure 24:**
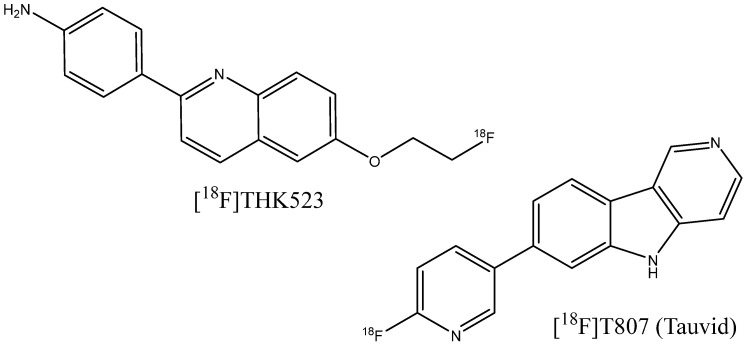
Structure of [^18^F]THK523 and [^18^F]T807.

The search for compounds targeting tau fibrils started from the screening of more than 2000 molecules in order to find strong tau binders with relatively low affinity to beta-amyloid fibrils [160]. Weak binding to Aβ is necessary, since neurofibrillary tangles colocalize with fibril plaques. The identified hits were lipophilic benzoxazole, benzimidazole, and quinolone derivatives, which were further optimized into PET imaging agent candidates [^18^F]THK-523 (Figure 24) [161,162]. However, this compound was reported to bind to Aβ plaques as well [163]. Exploration of a different series of compounds, including pyridoindole derivatives [163], led to the discovery of a promising candidate, [^18^F]T807 (Figure 24), which has now become an approved PET imaging agent taucipir, sold under the brand name Tauvid and also known as [^18^F]AV-1451. This compound was found to have a nanomolar affinity to tau fibrils, not to bind to Aβ, and to cross the blood–brain barrier efficiently [163].

**Figure 25:**
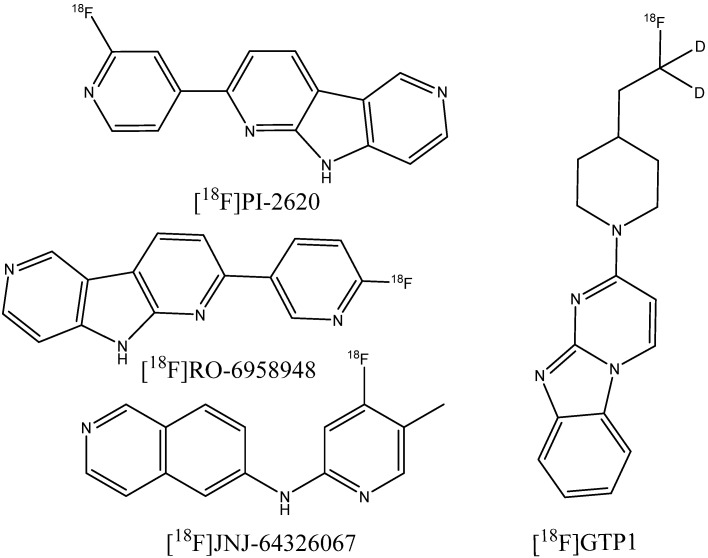
Structure of [^18^F]PI-2620, [^18^F]RO-6958948, [^18^F]JNJ-64326067, and [^18^F]GTP1.

However, flortaucipir and some other probe candidates from the THK family have shown significant off-target binding to monoamine oxidases A and B [164,165]. Thus, an additional requirement for the development of so-called second-generation tau PET imaging agents [166] is the low affinity of compounds to monoamine oxidases. A number of novel compounds have undergone investigations and clinical trials, including the aforementioned [^18^F]APN-1607, [^18^F]MK-6240 [167,168], [^18^F]PI-2620 [169], [^18^F]RO-948 [170,171], [^18^F]JNJ-067 [172], and [^18^F]GTP1 [173] (Figure 25). Some of the designed agents have also shown potential for the detection of non-AD tauopathies, including progressive supranuclear palsy [169,174,175] or frontotemporal dementia [176].

**Figure 26:**
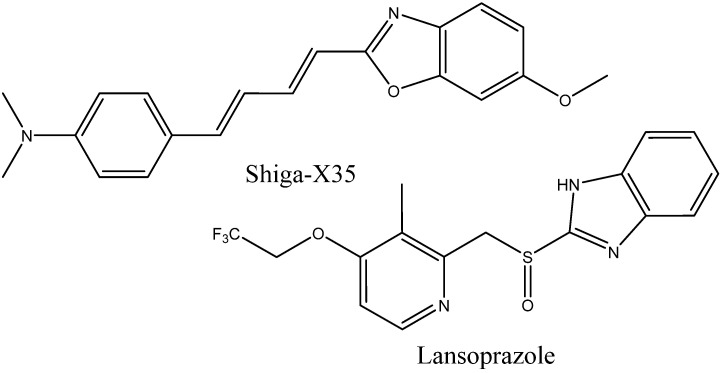
Structure of Shiga-X35 and lansoprazole.

Attempts to use MRI probes for tau imaging have recently been made. Tooyama et al. [49] synthesized the fluorine-containing NFT-binding compound Shiga-X35 (Figure 26), which still needs further optimization due to its low selectivity, which results in the presence of strong unwanted signals. Another promising compound is lansoprazole (Figure 26), which is a commercial fluorine-containing drug with a high affinity to tau fibrils [177]. Modification of the developed PET agents with fluorine-containing substituents in order to use them as MRI probes may also become a fruitful approach.

The development of the cryo-EM technique and its use for the structural analysis of fibrillar aggregates and their complexes opens the opportunity for the structure-based design of probes, including their optimization, for a particular pathogenic protein or disease-determined fibril conformation. An example of the currently available structure is the complex with tau protein fibrils [123], shown above in Figure 19.

#### 3.2.2. Probes for Visualization of Non-AD-Related Amyloid Deposits

**Figure 27:**
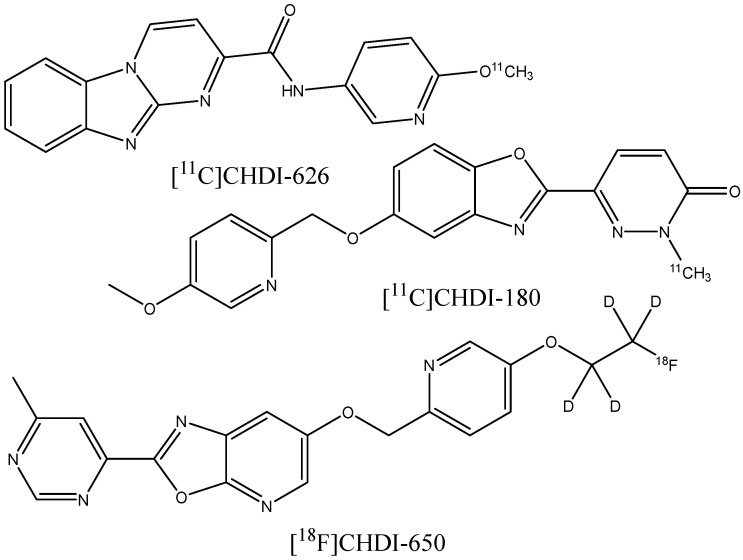
Structure of [^11^C]CHDI-626, [^11^C]CHDI-180, and [^18^F]CHDI-650.

At least some of the diagnostic agents developed for AD-related amyloid plaque imaging can also be successfully used for the visualization of fibrillar protein deposits accumulating in various organs, especially in the heart, due to the different amyloid diseases. It turned out to be possible to use Pittsburgh compound B [178,179,180] and the FDA-approved radiopharmaceuticals [^18^F]flutemetamol [181], [^18^F]florbetapir [182,183], and [^18^F]florbetaben [184] for PET diagnostics of systemic immunoglobulin light chain (AL) amyloidosis and hereditary transthyretin amyloidosis. In a recent series of works [185,186,187], the novel PET ligands [^11^C]CHDI-180, [^11^C]CHDI-626, and [^18^F]CHDI-650 (Figure 27) were designed to bind huntingtin protein fibrils in the brain in order to diagnose Huntington’s disease (HD). The design started from screening a collection of analogs of beta-amyloid binders, followed by an empirical exploration of the structure−activity relationships to increase the affinity and selectivity of binding to huntingtin fibrils relative to the Aβ fibrils in order to discriminate between HD and AD.

**Figure 28:**
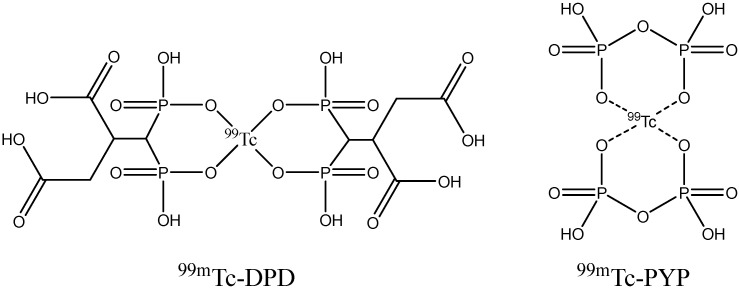
Structure of ^99m^Tc-DPD and ^99m^Tc-PYP.

The low selectivity of many fibril binders can be explained by the common structural features shared by amyloid fibrils of any origin, namely, the existence of a beta-sheet secondary structure, which is responsible for the interactions with molecular probes. Fluorescent indicators are also known to bind fibrils formed from different proteins [188,189]. It is likely that the MRI tracers discussed above also have some potential for visualization of non-AD amyloidoses. Development of the specific MRI agents particularly aimed at non-AD fibril deposits has not received the attention of researchers. One of the reasons is that these diseases are much rarer than AD. Moreover, scintigraphic diagnostic methods have been developed and applied in clinical practice, including ^123^I–SAP scans using the ^123^I-labeled serum amyloid P component, which binds to amyloid fibrils; ^99m^Tc-DPD or ^99m^Tc-PYP (Figure 28) scintigraphy using bone-seeking radiopharmaceuticals, which are thought to accumulate in amyloid deposits due to the high concentration of calcium ions [179,190]; and PET and SPECT antibody imaging methods [191,192]. These methods cannot be used to visualize amyloid structures in the brain, since the corresponding probes cannot cross the BBB.

MRI techniques that do not use any special chemical agents, such as native myocardial T1 mapping or steady-state free precession cine imaging for evaluating cardiac amyloidosis [193,194,195], are also emerging, but they are out of the scope of the present review.

### 3.3. Metal Complex-Based Contrast Agents

**Figure 29:**
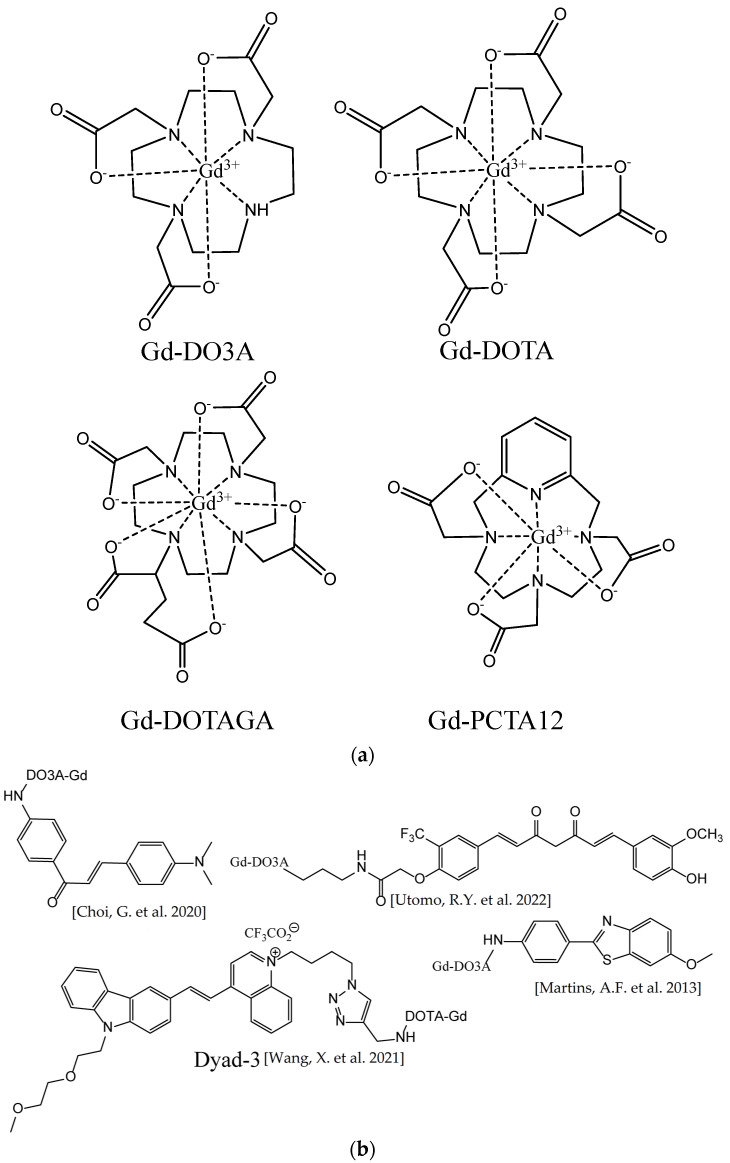
(**a**) Gadolinium contrast agents (non-fibril-targeted) tested in vivo for the ability to visualize amyloidoses. (**b**) Examples of targeted contrast agents binding β-amyloid plaques. Gadolinium complexes are coupled through—CO-NH- bonds, Literature references are given in brackets [210,211,212,213].

**Figure 30:**
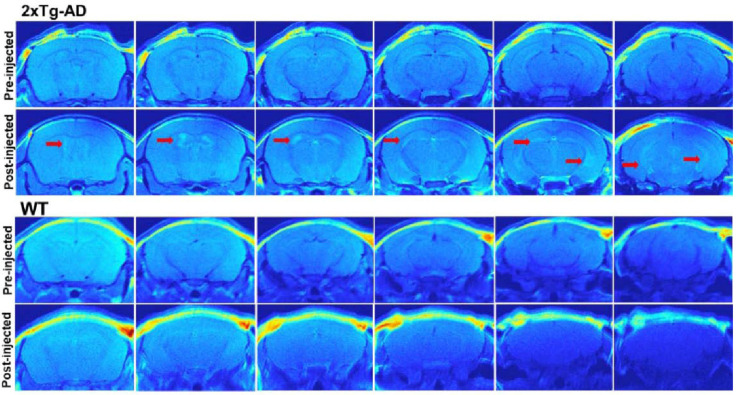
In vivo *T*_1_-weighted ^1^H MR images of the brains of transgenic AD model (2xTg-AD, **top**) and wild-type (WT, **bottom**) mice at different depths, before and after injection of Gd-DOTA-based amyloid-targeted contrast agent Dyad-3. Red arrows indicate the amyloid-rich regions. Adapted with permission from [211]. Copyright 2021 American Chemical Society.

There are various gadolinium complexes (Figure 29) [16,83,196] and manganese-based [197] contrast agents that have been applied to visualize different amyloidoses, including AD, in humans and animals, but do not have an affinity to the fibrillar structures. These agents are hydrophilic and distribute in the tissues surrounding the plaques, but cannot penetrate the hydrophobic amyloid deposits. This increases the imaging contrast of ^1^H MRI between the plaques and the tissues due to the relaxation rate enhancement of the spins of the water protons near paramagnetic metal ions. To further increase the sensitivity, metal-based targeted contrast agents that are able to bind fibrils were suggested [198]. Most of them were Gd^3+^ chelate complexes in which the ligand was covalently linked with a fibril-binding molecule. In the pioneering work of Poduslo et al. [199] and in a number of later studies, β-amyloid peptides or their fragments and derivatives were conjugated with gadolinium complexes [200,201,202] or superparamagnetic iron oxide nanoparticles (SPIONs) [203,204]. Alternatively, β-amyloid antibodies and their derivatives or fragments were conjugated [205,206,207,208,209]. Many recent studies have employed Gd^3+^ chelates or iron oxide nanoparticles covalently or noncovalently linked with organic ligands binding Aβ fibrils. Conjugates of a chalcone derivative with Gd-DO3A complex [210]; carbazole-based cyanine with Gd-DOTA complex [211]; curcumin and Pittsburgh compound B with Gd-DO3A [212]; N,N-dimethylated Pittsburgh compound B and its benzoxazole and stilbene analogs with Gd-PCTA12 and Gd-DOTAGA [83]; thioflavin T derivatives [213] with Gd-DO3A and its derivatives [214]; and curcumin [215] and DDNP carboxyl derivative [216] with SPIONs were synthesized and tested in vitro, ex vivo, and, in some cases, in animal models. Another example is the Congo Red molecule noncovalently bound to bovine serum albumin, which is itself covalently linked with the Gd-DTPA complex [217]. All of these conjugated ligands were known β-amyloid fibril binders, most of which had previously been tested as fluorescent or PET imaging probes or their close analogs. An example of in vivo *T*_1_-weighted ^1^H MR images of mouse brains is shown in Figure 30.

The ability of many fibril binders to slow down fibril growth in vitro [218] led to the positioning of some β-amyloid targeting metal complexes as theranostic agents suitable for both the detection and inhibition of amyloid aggregation [212,219]. As no fibrillation inhibitor has yet shown potential to treat AD in vivo, these statements should be considered cautiously.

The common problem of metal complexes and nanoparticles for brain MR imaging is their poor BBB permeability. The strategies to improve it include additional conjugation with lipophilic molecules such as putrescine [199,206], polymers like polyethylene glycol [203], cell-penetrating Tat-peptide [204], liposomes [220], or nanovehicles [221]. Some covalently linked hydrophobic molecules [210,211], peptides, and antibodies [208,222] have been reported to provide the ability of the agent to cross the BBB without further modifications.

Conjugation of a metal chelate also markedly reduces the binding of organic ligands to β-amyloid fibrils [198]. Thus, preliminarily designed and tested small organic molecules that strongly bind Aβ fibrils (discussed above in Section 3.1.2) should become the basis for further synthesis of the metal complexes with higher affinity.

**Figure 31:**
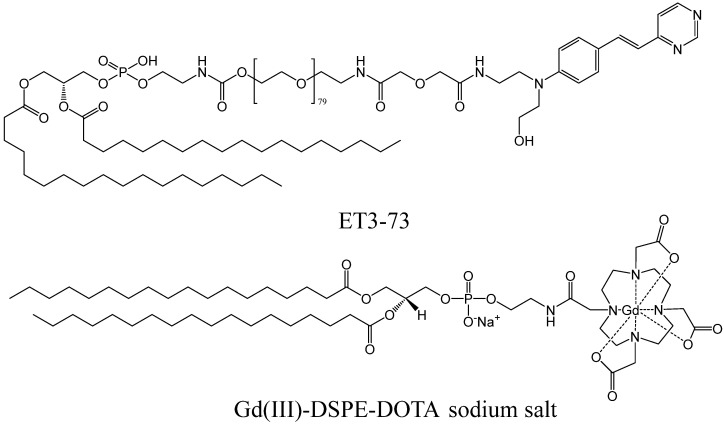
Key components of ADx-001 β-amyloid targeted contrast agent entering clinical trials [220].

No metal-based targeted contrast agents aiming at amyloid deposits have passed clinical trials yet. In December 2022, gadolinium-based contrast agent ADx-001 entered Phase 1 of clinical trials [223], which is to be completed in 2024. ADx-001 is an intravenously delivered liposome-based composition containing DSPE phospholipid-bound Gd-DOTA chelate and a fibril-binding conjugate of DSPE, PEG3500, and a styrylpyrimidine moiety (Figure 31) [220,224].

## 4. Conclusions

The MRI technique with amyloid-binding molecular probes presents a promising avenue for the early detection and monitoring of AD and some other amyloidoses. MRI is a widely available and clinically established method which has a high spatial resolution and does not involve the use of radioactive compounds. Over the past decade, several promising MRI probes that exhibit high affinity and specificity for amyloid deposits in preclinical models have been discovered. However, further studies are needed in order to optimize the design of these agents and to validate their applicability in vivo.

The major challenge is the low signal intensity of fluorinated MRI probes. This can be enhanced by increasing the number of fluorine atoms in its molecule. Unfortunately, this promotes off-target interactions with brain lipid components and decreases the aqueous solubility of the probes. Simultaneous introduction of a number of MRI-active fluorinated groups and polar fragments decreasing the hydrophobicity is possible, but leads to a larger molecular size of the probe, which would require special transport strategies to pass through the BBB.

Binding affinity of the probe to amyloid plaques can be provided by a variety of known molecular scaffolds. They contain conjugated aromatic fragments and double bonds and are able to adopt planar conformations. Recent breakthroughs in the determination of the atomic resolution structure of fibril aggregates open new possibilities for the computational structure-based design of diagnostic probes, including their optimization for a particular structure of fibrils deposited in the case of a certain pathological condition.

Alternatively, metal-based targeted contrast agents that are able to bind amyloid structures can be used to increase the imaging contrast of ^1^H MRI between the plaques and the tissues. The metal ions in these agents should be conjugated with organic amyloid-binding ligands.

Table 1 lists the most prominent MRI probe candidates considered in the present review with the ability to bind amyloid aggregates.

Further efforts aimed at the design, synthesis, and extensive testing of potential MRI probes are required for clinical translation.

## Figures and Tables

**Table 1:** Existing MRI probe candidates with the ability to bind amyloid aggregates.

Compound Names and Literature References	Target	Figure ^1^
For ^19^F imaging		
FSB [90]	Aβ	8
TFMB-2Et, TFMB-3Et [93]	Aβ	9
Shiga-Y5 [94,95]	Aβ	10
Shiga-X22 [67,96]	Aβ	10
Shiga-Y51 [97]	Soluble Aβ aggregates	10
7d [98]	Aβ	13
BSA-capped GQDs functionalized with hydrofluorinated glucose [99]	Aβ	–
Shiga-X35 [49]	Tau	26
Lansoprazole [177]	Tau	26
For ^1^H contrast imaging, metal-based		
Gd^3+^ chelates conjugated with Aβ binders [200,201,202,210,211,212,213,214]	Aβ	29b
SPIONs conjugated with Aβ binders [203,204]	Aβ	–
ADx-001 [220,224]	Aβ	31

^1^ The number of the figure showing the probe’s structure in the present paper.
